# Integrated MALDI-MSI and UHPLC-OE-MS for Spatial Visualization and Biosynthetic Pathway Elucidation of Bioactive Metabolites in *Lilium lancifolium* Thunb.

**DOI:** 10.3390/molecules31111820

**Published:** 2026-05-25

**Authors:** Qibo Deng, Zhihui Wang, Jiajia Ji, Minsi Xie, Qiaozhen Tong, Kunlai Sun, Qinghua Peng, Zhiying Yuan

**Affiliations:** 1 School of Pharmacy, Hunan University of Chinese Medicine, Changsha 410208, China; 20243795@stu.hnucm.edu.cn (Q.D.); 004595@hnucm.edu.cn (Z.W.); 20243800@stu.hnucm.edu.cn (J.J.); 20233797@stu.hnucm.edu.cn (M.X.);; 2 Academic Department, Beijing Jiansheng Pharmaceutical Co., Ltd., Beijing 101520, China; 3 Fresh Herbal Medicine Academic Committee of China Cancer Foundation, Beijing 101520, China; 4 School of Traditional Chinese Medicine, Hunan University of Chinese Medicine, Changsha 410208, China

**Keywords:** *Lilium lancifolium* Thunb., MALDI-MSI, UHPLC-OE-MS, steroidal saponins, phenolic acid glycerides, biosynthetic pathway

## Abstract

*Lilium lancifolium* Thunb. is an important economic crop widely cultivated and traded across Asia and has significant pharmacological activity. Despite decades of research on their chemical composition, the spatial distribution patterns of characteristic secondary metabolites within the bulbs remain poorly understood. In this study, we used matrix-assisted laser desorption/ionization mass spectrometry imaging (MALDI-MSI) technology to characterize and spatially visualize multiple metabolites within the bulb for the first time. Additionally, ultra-high-performance liquid chromatography-Orbitrap Exploris mass spectrometry (UHPLC-OE-MS) was used to obtain comprehensive metabolite information from the bulbs. Using spatial metabolomics, we successfully identified nine steroidal saponins, three phenolic acid glycerides, and six other metabolites. Subsequently, we analyzed the spatial distribution of steroidal saponins and phenolic acid glycerides, which are key bioactive components. The analysis revealed that most of the steroidal saponins and phenolic acid glycerides, such as deacylbrownioside and regaloside A, exhibited a similar distribution pattern, mainly being enriched in the outer regions (A2, B2) and basal regions (B1, B2) on an individual scale. Further metabolomic and Kyoto Encyclopedia of Genes and Genomes (KEGG) pathway enrichment analyses indicated that 11 substances detected in the bulbs, including diosgenin, phenylalanine, and acetyl-CoA, were jointly associated with 39 metabolic pathways, including “phenylpropanoid biosynthesis” and “terpenoid backbone biosynthesis”. Based on the above findings, we propose biosynthetic pathways and accumulation patterns of steroidal saponins and phenolic acid glycerides in bulbs. This study provides a basis for precise resource utilization of *L. lancifolium* bulbs and a methodology to elucidate the biosynthesis of plant metabolites.

## 1. Introduction

Lilies are important crops with a broad industrial value regarding medicinal, food, and ornamental applications. According to the 2025 edition of the Chinese Pharmacopoeia [1], the dried bulbs of three plant species, namely, *Lilium lancifolium* Thunb., *Lilium brownii* F.E. Brown var. *viridulum* Baker, and *Lilium pumilum* DC., are the official sources of lily medicinal herbs. As documented in the Compendium of Materia Medica (Ben Cao Gang Mu), China’s ancient medical canon, the core efficacy of the lily is to “nourish yin and moisten the lungs, clear the heart and calm the nerves”, indicating its ability to relieve dry cough in autumn, and improve symptoms such as insomnia, excessive dreaming, and mental confusion [2]. As one of the most significant medicinal and edible homologous herbs in China, the annual output of Chinese lilies exceeds 100,000 tons [3]. *L. lancifolium* is the main lily variety circulating in the market, with typical morphological characteristics such as clusters of dark brown bulbils in the leaf axils and densely distributed purple striped markings on the stem surface. It is primarily distributed in Longshan of Hunan, Yixing of Jiangsu, and Guangxi. Additionally, there are some wild resources. Longshan County is the core production area of *L. lancifolium* in China. It holds the status of a National Geographical Indication for its lily products.

Lilies hold a significant position in health food development and clinical dietary therapy due to their suitability for large-scale cultivation and their rich content of bioactive components, such as polysaccharides and steroidal saponins in bulbs, embodying the core concept of “food and medicine sharing the same origin”. In recent years, the identification of secondary metabolites in lilies and the exploration of their pharmacological mechanisms have become popular research areas in food science and natural medicine. Existing research indicates that *L. lancifolium* contains a complex array of bioactive compounds, primarily steroidal saponins, phenolic acid glycerides, polysaccharides, and alkaloids. These components have diverse pharmacological activities, including antidepressant, antioxidant, anti-inflammatory, neuroprotective, and immunomodulatory effects, demonstrating their potential value in antitumor and anti-aging research [4,5,6,7]. Owing to their unique chemical structures and broad biological activities, steroidal saponins and phenolic acid glycerides are considered characteristic functional components of *Lilium* species, and thus, remain under continuous research scrutiny. Previous studies successfully identified a series of representative compounds from *Lilium* species, including typical steroidal saponins, such as deacylbrownioside [8] and dioscin [9], as well as typical phenolic acid glycerides, such as regalosides A and B [10]. Regaloside A was confirmed to be the primary phenolic acid glyceride component of *L. lancifolium* bulbs [11], which feature a characteristic phenylpropanoid skeleton. However, despite the elucidation of the chemical structures of these components, a critical gap remains in the current research paradigm: we lack an intuitive and systematic understanding of the in situ distribution and spatial accumulation patterns of these key bioactive compounds, particularly steroidal saponins and phenolic acid glycerides, in mature bulb tissues.

The spatial distribution patterns of key metabolites in mature *L. lancifolium* bulbs are crucial for precisely mapping the biosynthetic and accumulation mechanisms of secondary metabolites, dynamically correlating the physiological functions of bioactive compounds in functional foods with their in situ locations, and enabling targeted utilization of this medicinal and edible dual-purpose resource. Currently, plant metabolite analysis primarily relies on chromatography–mass spectrometry (MS) technologies, including high-performance liquid chromatography (HPLC), gas chromatography (GC), gas chromatography–mass spectrometry (GC-MS), and liquid chromatography–mass spectrometry (LC-MS) [12,13]. These techniques offer high sensitivity and robust identification capabilities, making them widely used. For instance, offline two-dimensional liquid chromatography has revealed an abundance of phenylpropanoid derivatives and steroidal saponins in *L. lancifolium* and *L. brownii viridulum* [14]. Multiplex GC-MS and UPLC-MS/MS techniques have been used to analyze volatile and non-volatile metabolites in *Mimusops caffra* leaves [15]. Notably, the application of advanced technologies, such as ultra-high-performance liquid chromatography/quadrupole time-of-flight mass spectrometry (UHPLC-QTOF-MS) and UHPLC-OE-MS, has significantly enhanced the resolution, sensitivity, and throughput of metabolite detection [16], thereby enabling metabolomics to interpret chemical information within complex biological samples at a deeper level. However, all these methods involve extraction followed by detection, and the necessary pretreatment steps inevitably disrupt the original tissue structure. This imposes a fundamental limitation, as they cannot preserve or provide information on the in situ spatial distribution of metabolites within the native tissue microenvironment. Although combining omics data with machine learning enables the efficient screening of biomarkers in traditional Chinese medicinal materials, thereby linking the material quality to efficacy, this association remains based on statistical inference from macroscopically homogenized data, which lacks direct visual–spatial evidence. Therefore, the development of novel, in situ, nondestructive, highly sensitive, and rapid spatial analysis techniques is crucial.

In recent years, mass spectrometry imaging (MSI) has achieved significant breakthroughs as a powerful spatially resolved analytical tool in fields such as food science, plant biology, and medical research [17,18,19]. This technology enables the direct scanning of biological tissue sections without relying on labeling or complex extraction. Two-dimensional ion density distribution maps of the compounds within their native tissue contexts were generated by detecting ion signals at specific mass-to-charge ratios (*m*/*z*). MSI simultaneously captures the composition, relative abundance, and precise spatial distribution of multiple compounds within tissues, providing irreplaceable visual evidence for biomarker discovery and in situ drug metabolism monitoring. Compared with LC-MS, which relies on tissue homogenate extraction, MSI can directly analyze tissue sections and obtain compound information in situ, effectively avoiding the loss of trace substances during the extraction process [20]. It is worth noting, however, that the micro-spatial focus of MSI naturally introduces an analytical trade-off, as the absence of continuous chromatographic separation and the practical challenges of deploying high-throughput tandem mass spectrometry at the imaging level can increase the complexity of definitive structural annotation for closely related molecular analogues [21].

MSI techniques include matrix-assisted laser desorption/ionization (MALDI), desorption electrospray ionization (DESI), and secondary ion mass spectrometry (SIMS). MALDI-MSI has become the most commonly used and effective technique for visualizing plant metabolites owing to its suitable spatial resolution and soft ionization characteristics across a wide mass range [22,23]. Its applications have demonstrated significant value. For instance, one study used MALDI-MSI to visualize the spatial distribution of metabolites, including alkaloids and sesquiterpenoids in *Dendrobium nobile* stems [24]. Another study tracked the spatial and temporal regulation patterns of metabolites in the rhizomes, primary roots, branched roots, and fibrous roots of *Panax notoginseng*, as well as in the microregions of the ligamentous part, xylem, medulla, and cork during the steaming process, providing new insights into the mechanisms of medicinal material processing [25]. Furthermore, previous research [26] combined LC-MS with MALDI-MSI to construct the spatiotemporal metabolic profiles of black and yellow-brown *tartary buckwheat* achenes at three important developmental stages. This study revealed the specific spatial distribution patterns of flavonoid components during achene development and elucidated the potential regulatory mechanisms of flavonoid components in embryo development and pericarp color formation in *tartary buckwheat* achenes. Recent technological advances have enabled the development of matrix-assisted laser desorption/ionization mass spectrometry imaging-based plant tissue microarrays (MALDI-MSI-PTMA) to overcome limitations in detecting internal metabolites within plant tissues (e.g., cuticle layers and cell walls) [19]. This approach significantly enhances the detection throughput and efficiency, demonstrating the ongoing optimization and formidable potential of MALDI-MSI technology for analyzing complex plant samples.

In this study, we focused on metabolites and their relative contents in mature *L. lancifolium* bulbs, emphasizing the visualization and spatial distribution of steroidal saponins and phenolic acid glycerides while elucidating their biosynthetic pathways. The results show that MALDI-MSI directly visualized the fine spatial distribution patterns of steroidal saponins and phenolic acid glycerides in *L. lancifolium* bulbs. Combined with UHPLC-OE-MS, this approach enabled an in-depth analysis of the biosynthetic pathways. This integrated strategy established a high-sensitivity, spatially resolved analytical framework that surpassed traditional bulk analysis by deciphering the intricate metabolic heterogeneity within complex tissues. Beyond mere visualization, it provided unprecedented insights into the spatial orchestration of specialized metabolites, effectively bridging the gap between localized chemical accumulation and in situ physiological roles. These findings offer a robust scientific basis for elucidating the ecological functions of bioactive constituents, ultimately facilitating the optimized utilization of high-value medicinal and edible crops.

## 2. Results and Discussion

### 2.1. Metabolic Profiling of L. lancifolium Bulb Using MALDI-MSI Analysis

**Figure 1:**
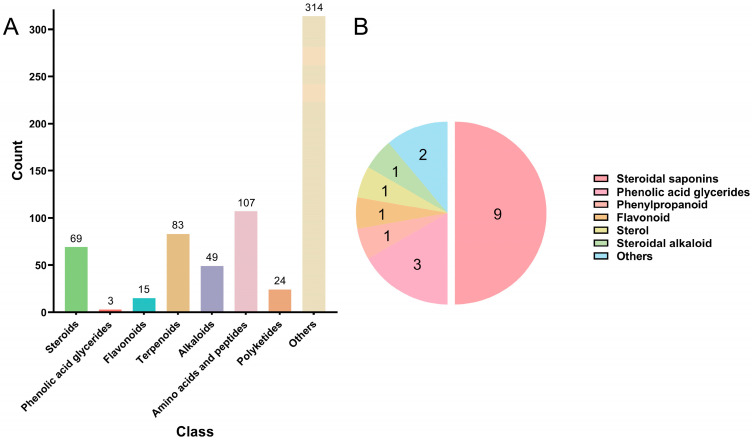
Overall MALDI-MSI metabolite map: (**A**) preliminary database matched metabolite profiles; (**B**) secondary database exactly matched metabolite profiles.

As a plant with both medicinal and edible value, *L. lancifolium* has been studied for many years, and its chemical composition has been thoroughly analyzed. However, a research gap remains regarding the spatial distribution of metabolites in bulb tissues. In this study, spatial metabolomics was used to analyze *L. lancifolium* bulb for the first time. A total of 664 metabolites were identified by comparison with the natural product database of RefMetaDB and laboratory standards, including 69 steroids, 3 phenolic acid glycerides, 15 flavonoids, 83 terpenoids, 49 alkaloids, 107 amino acids and short peptides, 24 polyketides, and 314 other types of compounds (Figure 1A).

**Figure 2:**
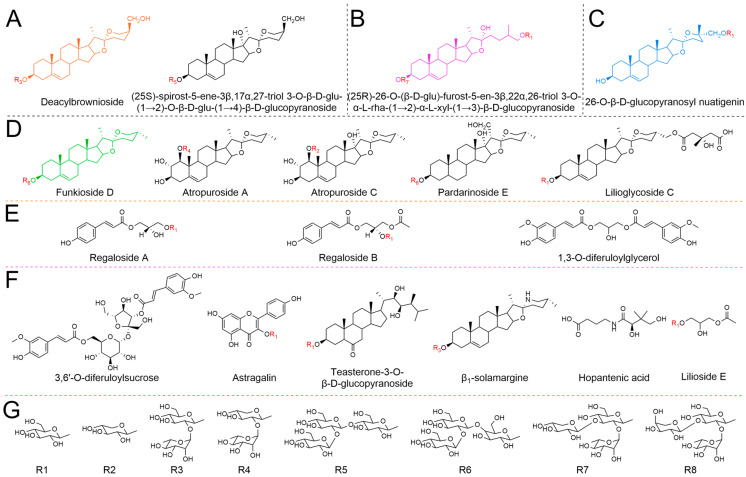
The Chemical structural formulas of 18 substances identified using MALDI-MSI: (**A**) spirostanol saponins; (**B**) furostanol saponins; (**C**) pseudospirostanol saponins; (**D**) isospirostanol saponins; (**E**) phenolic acid glycerides; (**F**) other type substances; (**G**) substituent groups.

Because the previously used database mainly targeted conventional plant metabolites, we adopted a more precise matching strategy to further improve the matching degree of the specific metabolites of *L. lancifolium*. First, we downloaded a database of specific metabolites involved in the biosynthetic pathways of the genus *Lilium* from the official Reaxys website. Next, in positive-ion mode, we matched five adduct ion forms—[M + H]^+^, [M + Na]^+^, [M + K]^+^, [M + NH_4_]^+^, and [M + H-H_2_O]^+^—while controlling the *m*/*z* error range to within ±12 ppm during the matching process. This approach enabled accurate analysis of the spatial metabolomics data. Through a systematic comparison of the spatial metabolomics data with the *Lilium* database and the exclusion of uncertain substances to ensure structural certainty, we ultimately identified 18 distinct compounds. This optimized protocol significantly enhanced the accuracy and reliability of *L. lancifolium* metabolite identification. Figure 1B shows that the 18 identified substances comprised 9 steroidal saponins, 3 phenolic acid glycerides, 1 phenylpropanoid, 1 flavonoid, 1 sterol, 1 steroidal alkaloid, and 2 others. Detailed information on the identified substances (class, chemical name, formula, mass error, and adduct forms) is presented in the Supplementary Materials. The results indicate that steroidal saponins and phenolic acid glycerides are the major active components in *L. lancifolium*, which is consistent with previous reports identifying these compounds as important bioactive constituents of this species [27]. Based on the structural characteristics of the core steroidal skeleton, the identified steroidal saponins were systematically classified into four categories: spirostanol saponins (deacylbrownioside, (25S)-spirost-5-ene-3β,17α,27-triol 3-O-β-D-glu-(1→2)-O-β-D-glu-(1→4)-β-D-glucopyranoside) (Figure 2A), furostanol saponins ((25R)-26-O-(β-D-glu)-furost-5-en-3β,22α,26-triol 3-O-α-L-rha-(1→2)-α-L-xyl-(1→3)-β-D-glucopyranoside) (Figure 2B), pseudospirostanol saponins (26-O-β-D-glucopyranosyl nuatigenin) (Figure 2C), and isospirostanol saponins (funkioside D, atropuroside A, atropuroside C, pardarinoside E, lilioglycoside C) (Figure 2D). In addition, three classes of phenolic acid glycerides were detected in this study, namely, regalosides A and B, and 1,3-O-diferuloylglycerol (Figure 2E). Meanwhile, six compounds were classified as other types, namely, 3,6′-O-diferuloylsucrose, astragalin, teasterone-3-O-β-D-glucopyranoside, β_1_-solamargine, hopantenic acid, and lilioside E (Figure 2F).

Steroidal saponins in *L. lancifolium* exhibit a wide range of pharmacological effects. Regarding antitumor activity, steroidal saponins exert their effects through multiple mechanisms, including the induction of tumor cell apoptosis, inhibition of tumor migration, and blockade of the cell cycle. To date, over 40 steroidal saponins with antitumor activity have been reported [28]. In addition, steroidal saponins also possess anti-inflammatory, antibacterial, immunomodulatory, neuroprotective, and hypoglycemic activities [29]. On the other hand, phenolic acid glycerides also demonstrate significant biological activities. Studies showed that phenolic acid glycerides exhibit good antioxidant activity by effectively scavenging free radicals, along with antibacterial and anti-inflammatory effects [30]. Notably, the glycosylated forms of phenolic acids generally possess higher bioavailability, and their activities are dependent on the structure of the aglycone, as well as the position and type of glycosidic linkages [31]. Because direct tissue profiling primarily captures MS1 signals and lacks baseline chromatographic separation and MS/MS spectra, it is inherently unable to resolve stereochemical isomers [32]. As highlighted by García-Rojas, N. S. et al. [33], annotations from direct profiling platforms generally fall within Level 2 or Level 3 confidence, whereas definitive Level 1 identification requires authentic standards under identical conditions. Accordingly, within these analytical constraints, we have adopted a conservative annotation strategy throughout this study.

### 2.2. Visualization and Spatial Distribution of Different Partitions and Metabolites in L. lancifolium Bulb

The bulb serves as the primary nutrient storage and reproductive organ in *L. lancifolium*. It has a unique appearance; is ovoid in shape; and comprises multiple layers of white, broadly ovate, fleshy scales arranged in an imbricate pattern. Based on the morphological characteristics and previous research reports [34], we divided a single bulb scale into four characteristic regions: apical middle (A1), apical edge (A2), basal middle (B1), and basal edge (B2), as depicted in Section 3.1. This partitioning method provides a clear spatial localization reference for subsequent research and aids in analyzing the specific distribution patterns and biological significance of secondary metabolites such as steroidal saponins and phenolic acid glycerides in the bulb.

#### 2.2.1. Visualization and Spatial Distribution of Steroidal Saponins in *L. lancifolium* Bulb

**Figure 3:**
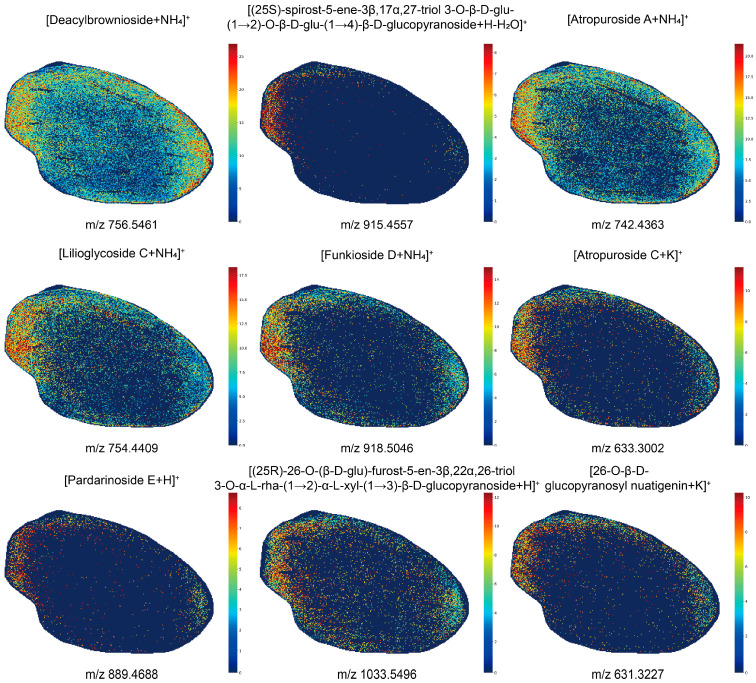
Spatial distribution maps of steroidal saponins in *L. lancifolium* bulb using MALDI-MSI.

Through spatial metabolomics analysis, we successfully visualized and identified nine steroidal saponin compounds with diverse structures in the tissue sections of *L. lancifolium* bulb (Figure 2A–D). Figure 3 shows that these metabolites were distributed across four characteristic regions (A1, A2, B1, B2) of a single bulb but exhibited distinct regional enrichment differences. Specifically, deacylbrownioside, atropuroside A, and lilioglycoside C showed relatively high spatial distribution densities. In contrast, (25S)-spirost-5-ene-3β,17α,27-triol 3-O-β-D-glu-(1→2)-O-β-D-glu-(1→4)-β-D- glucopyranoside, funkioside D, atropuroside C, pardarinoside E, and (25R)-26-O-(β-D-glu)-furost-5-en-3β,22α,26-triol 3-O-α-L-rha-(1→2)-α-L-xyl-(1→3)-β-D-glucopyranoside, along with 26-O-β-D-glucopyranosyl nuatigenin exhibited low spatial distribution densities. Notably, we observed a key spatial distribution pattern: except for deacylbrownioside, the other eight steroidal saponins exhibited higher enrichment in the basal regions (B1, B2) of the bulb than in the apical regions (A1, A2). Meanwhile, we also found that the spatial distribution densities of these nine steroidal saponins were generally higher in the outer regions (A2, B2) of the bulb than in the central regions (A1, B1). This precise basipetal and peripheral spatial distribution pattern strongly suggests that steroidal saponin accumulation is not random but is strictly regulated by their physiological functions and biosynthetic transport pathways. We hypothesized that basal enrichment might be related to the role of the mature bulb as a storage organ, where high starch reserves [35] likely provide ample carbon skeletons and precursors for steroid saponin biosynthesis. Conversely, such peripheral enrichment presents a plausible chemical defense barrier, representing a putative plant strategy of allocating defensive constituents to peripheral tissues [36,37]. However, the definitive physiological role of this spatial compartmentalization remains speculative pending direct functional validation. Notably, the pattern of steroidal saponin accumulation in specific regions of underground organs observed in this study exhibited inherent spatial consistency with the macro-level trend reported in the literature [38,39], where such compounds tend to be enriched in plant underground organs, such as glycyrrhizin in *Glycyrrhiza*. This consistency is further supported by reports of saikosaponins migrating from the pericycle and primary phloem toward the vascular cambium and secondary phloem during development in *Bupleurum chinense* roots [40], exhibiting similar directionality. Collectively, these findings support the critical hypothesis that steroidal saponins undergo conserved, highly organized, and spatially specific transport within plants. Furthermore, it should be noted that the embedded bulb samples in this study were stored at −80 °C. This condition maintains starch stability more effectively than conventional refrigeration [41], thereby maximally preserving the in situ distribution information of metabolites at the moment of death. This enhances the reliability of the observed spatial distribution patterns.

#### 2.2.2. Visualization and Spatial Distribution of Phenolic Acid Glycerides in *L. lancifolium* Bulb

**Figure 4:**
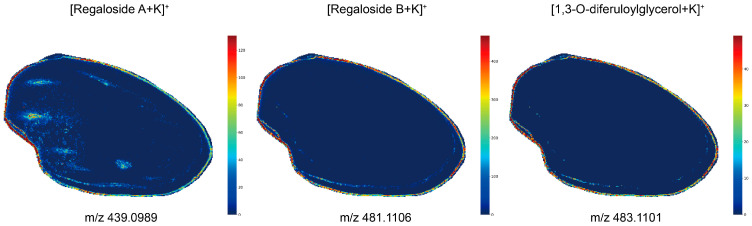
Spatial distribution maps of phenolic acid glycerides in *L. lancifolium* bulb using MALDI-MSI.

Based on spatial metabolomic analysis, we further visualized the spatial distribution of another class of characteristic secondary metabolites in *L. lancifolium* bulb (Figure 4). The results indicated that three substances—regalosides A and B, and 1,3-O-diferuloylglycerol—were primarily enriched in the outer regions (A2, B2) of the bulb and exhibited higher accumulation levels in the basal regions (B1, B2) than in the apical regions (A1, A2). Notably, the abundance of regalosides A and B far exceeded that of 1,3-O-diferuloylglycerol, and the overall signal intensities of all three compounds in the outer regions (A2, B2) were higher than those of the coexisting steroidal saponins.

We observed that the spatial distribution patterns of these phenolic acid glycerides were highly consistent with those of the previously noted steroidal saponins. This suggests that, despite divergent biosynthetic pathways, different classes of secondary metabolites in *L. lancifolium* bulb may be regulated by common high-level transcriptional regulation or transport mechanisms, leading to their coordinated localization within specific physiological compartments. For instance, in *Salvia miltiorrhiza* roots, phenolic acids are primarily enriched in the outer periderm [42]. Thus, our findings corroborate this literature, collectively revealing that diverse medicinal plants may follow a conserved “peripheral enrichment” strategy in the spatial distribution of phenolic acids within tissues. This suggests that such spatial distribution patterns may hold a universal biological significance for the survival and function of underground plant organs.

#### 2.2.3. Visualization and Spatial Distribution of Other Types of Substances in *L. lancifolium* Bulb

**Figure 5:**
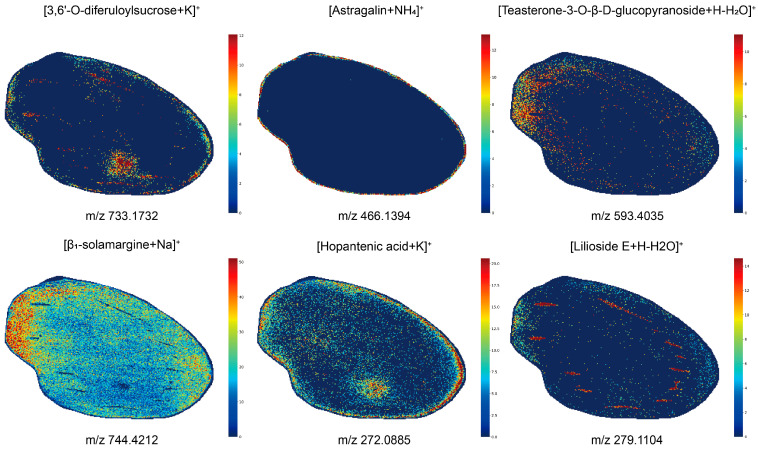
Spatial distribution maps of other substance types in *L. lancifolium* bulb using MALDI-MSI.

Through spatial metabolomics analysis, we also detected other metabolites, including the phenylpropanoid 3,6′-O-diferuloylsucrose, the flavonoid astragalin, the sterol teasterone-3-O-β-D-glucopyranoside, and the steroidal alkaloid β_1_-solamargine, as well as hopantenic acid and lilioside E. Spatial visualization via MALDI-MSI revealed that 3,6′-O-diferuloylsucrose, teasterone-3-O-β-D-glucopyranoside, β_1_-solamargine, hopantenic acid, and lilioside E were all distributed across the four regions (A1, A2, B1, B2) of the bulb, albeit with distinct enrichment patterns (Figure 5). Astragalin was predominantly enriched in the outer regions (A2, B2). 3,6′-O-diferuloylsucrose and hopantenic acid exhibited higher spatial distribution densities in the apical regions (A1, A2) and outer regions (A2, B2) compared with the basal regions (B1, B2) and the central regions (A1, B1). In contrast, teasterone-3-O-β-D-glucopyranoside and β_1_-solamargine showed higher accumulation in the basal regions (B1, B2) than in the top regions (A1, A2).

### 2.3. UHPLC-OE-MS Metabolomics Analysis

#### 2.3.1. Overall Metabolite Profile

**Figure 6:**
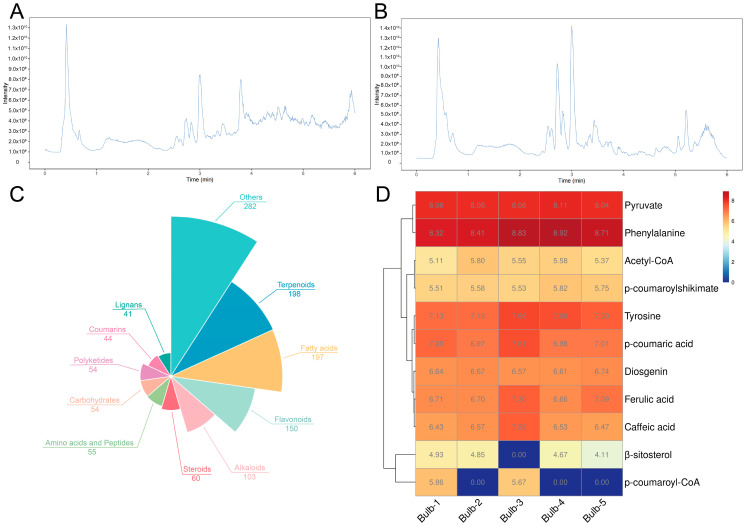
Metabolites in *L. lancifolium* bulbs were detected using UHPLC-OE-MS (n = 5): (**A**) total ion chromatogram in positive-ion mode; (**B**) total ion chromatogram in negative-ion mode; (**C**) overall metabolite profile using UHPLC-OE-MS; (**D**) cluster heatmap of 11 key precursor substances in bulbs.

Comprehensive metabolic analysis of the bulbs of *L. lancifolium* was conducted using non-targeted metabolomics via UHPLC-OE-MS. Data were collected in both positive- and negative-ion modes, and the corresponding total ion chromatograms (TICs) were plotted in Figure 6A,B. Comparative analysis revealed that the chromatographic peak information of the positive- and negative-ion TICs was highly complementary. Substances with different ionization characteristics exhibited optimal responses in specific modes, thereby effectively broadening the polarity range and chemical diversity of the detected substances. When the mass error was controlled to < 5 ppm, 1238 metabolites were initially identified (Figure 6C). These metabolites covered the main secondary metabolite categories, including 198 terpenoids, 197 fatty acids, 150 flavonoids, 103 alkaloids, 60 steroids, 55 amino acids and peptides, 54 carbohydrates, 54 polyketides, 44 coumarins, 41 lignans, and 282 others.

Based on metabolomic analysis, we detected 11 metabolites, including phenylalanine, acetyl-CoA, and diosgenin, serving as precursors for the biosynthetic pathways of steroidal saponins and phenolic acid glycerides in *L. lancifolium* bulbs. The key precursor substances for the synthesis of steroidal saponins included four substances: pyruvate, diosgenin, acetyl-CoA, and β-sitosterol. The key precursor substances for the synthesis of phenolic acid glycerides included seven substances, namely, phenylalanine, tyrosine, ferulic acid, caffeic acid, p-coumaric acid, p-coumaroyl-CoA, and p-coumaroylshikimate. To visually compare the relative abundances of these precursor substances in the bulbs, a clustered heatmap of different metabolites was drawn, where a redder color indicates a higher content and a bluer color indicates a lower content (Figure 6D). The analysis of precursor compounds provides key insights into the directionality of secondary metabolic flux in *L. lancifolium* bulbs. In the biosynthetic pathway of steroidal saponins, the abundance of the pyruvate precursor exceeded that of acetyl-CoA. This phenomenon suggests that the mevalonate (MVA) pathway in the bulb cytoplasm may dominate the supply of isopentenyl pyrophosphate (IPP) to construct steroid skeletons. This inference aligns with reports in the literature [43], indicating that the MVA pathway localized in the cytoplasm and mitochondria tends to supply more IPP for triterpenoid and steroid compound synthesis than the methylerythritol phosphate (MEP) pathway in the plastids. Concurrently, in the phenolic acid glyceride synthesis pathway, we detected lower levels of p-coumaroyl-CoA and p-coumaroylshikimate intermediates. This likely indicates that these key node compounds are rapidly converted in bulb tissues, with their metabolic flux efficiently directed downstream for the synthesis of phenolic glyceride compounds, such as regaloside A, or other coenzymes.

#### 2.3.2. KEGG Analysis

**Figure 7:**
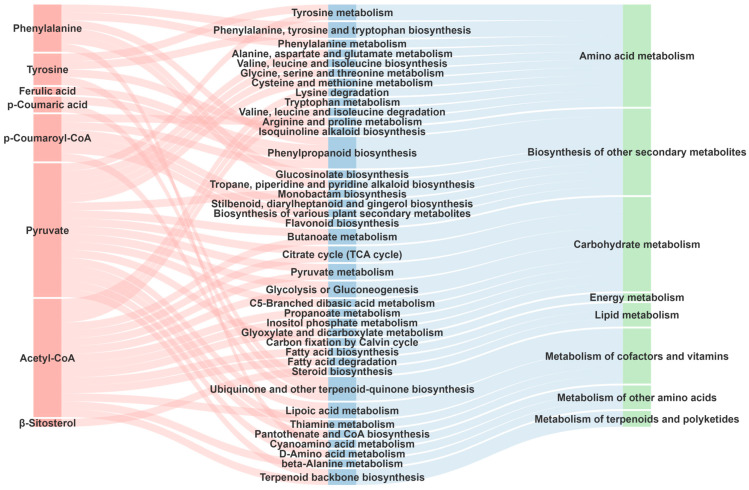
KEGG enrichment pathway analysis of 11 key precursor substances. The names of chemical substances are on the left, KEGG pathways are in the middle, and KEGG Subclass secondary classifications are on the right.

Using MetaboAnalyst 6.0, a KEGG pathway enrichment analysis was performed to analyze the metabolic pathways and physiological processes involved in these 11 key precursor substances in *L. lancifolium* bulbs (Figure 7). The KEGG annotation results indicate that these substances were involved in a total of 39 KEGG pathways, including “phenylpropanoid biosynthesis”, “terpenoid backbone biosynthesis”, and “flavonoid biosynthesis”. According to the KEGG Subclass secondary classification system, these pathways can be classified into eight major categories, such as “amino acid metabolism”, “biosynthesis of other secondary metabolites”, and “metabolism of terpenoids and polyketides”. The research found that four precursor substances—ferulic acid, p-coumaric acid, p-coumaroyl-CoA, and phenylalanine—were involved in the “phenylpropanoid biosynthesis” pathway in the KEGG database. According to the secondary classification, they belong to the “biosynthesis of other secondary metabolites”. Among these, p-coumaroyl-CoA serves as the core node and is widely involved in the “biosynthesis of various plant secondary metabolites”. Acetyl-CoA and pyruvate play crucial roles in the “terpenoid backbone biosynthesis” pathway by jointly constructing the basic skeleton of terpenoids and providing precursors for the synthesis of steroidal substances. As shared precursors, phenylalanine and tyrosine are successively converted into intermediates such as p-coumaric acid and ferulic acid via the “phenylalanine, tyrosine and tryptophan biosynthesis” and “phenylpropanoid biosynthesis” pathways, and ultimately, generate phenolic acid glycerides. Among them, p-coumaroyl-CoA serves as a key branching node, directly bridging the “phenylpropanoid metabolism” and the synthesis pathway of phenolic acid glycerides. Pyruvate and acetyl-CoA provide the carbon skeleton for the synthesis of steroidal saponins. Pyruvate is involved in “glycolysis or gluconeogenesis” and “pyruvate metabolism” to generate acetyl-CoA. The latter enters the “terpenoid backbone biosynthesis” pathway, and through the “steroid biosynthesis” pathway, steroidal precursors are generated, ultimately forming steroidal saponins, such as diosgenin. Notably, acetyl-CoA also provides energy and cofactor support for two metabolic pathways through the “fatty acid biosynthesis/degradation” pathway, highlighting its status as a metabolic hub. The metabolic association map constructed in this study reveals the central role of key precursors in the synthesis of steroidal saponins and phenolic acid glycerides in *L. lancifolium* bulbs.

### 2.4. Metabolic Pathways for the Biosynthesis of Steroidal Saponins in L. lancifolium Bulb

**Figure 8:**
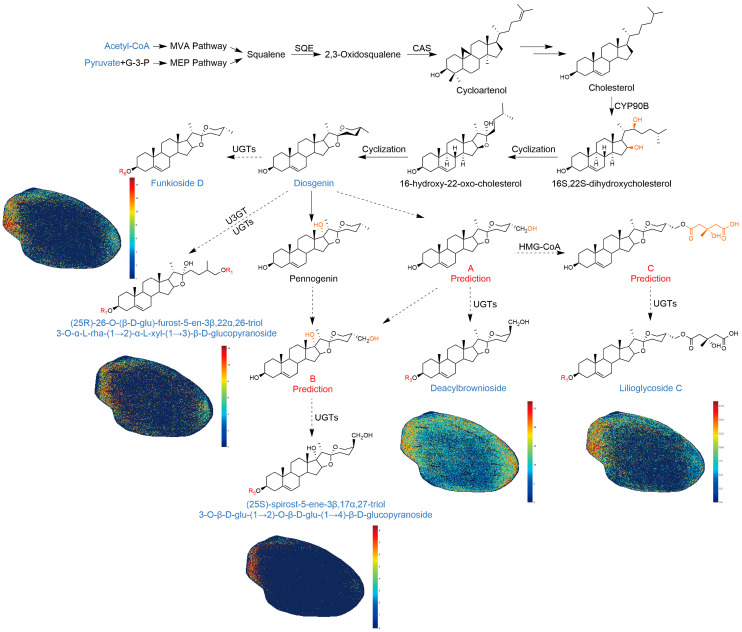
A metabolic pathway for the biosynthesis of steroidal saponins in *L. lancifolium* bulb is proposed based on the integrated analysis of MALDI-MSI and UHPLC-OE-MS. SQE, squalene epoxidase; CAS, cycloartenol synthase; CYP90B, cholesterol C22-monohydroxylase; UGTs, UDP-glu-cosyltransferase; U3GT, 3-O-sterolglycosyltransferase; HMG-CoA, 3-hydroxy-3-methylglutaryl-CoA. The solid black arrows indicate the biosynthetic pathways reported in the literature, while the dashed arrows represent the pathways we propose. (A–C) are the predicted skeletons of proposed intermediates. Substances labeled in blue font are detected in the present study, while those labeled in black font are not detected in this study.

Steroidal saponins are important components in *L. lancifolium* bulb and have anti-inflammatory, antioxidant, cholesterol-lowering, anti-thrombotic, and other biological activities [44,45]. In *Lilium* plants, steroidal saponins are a class of structurally complex secondary metabolites consisting of a polycyclic steroidal nucleus parent linked to a sugar moiety, and their biosynthesis involves a multi-step enzymatic pathway. Preliminary research has been conducted on the synthesis of this class of compounds. Certain key enzymes and their encoding genes have been identified, including squalene epoxidase (SQE), cycloartenol synthase (CAS), cytochrome P450 proteins (CYPs), UDP-glucosyltransferase (UGTs), and 3-O-sterol glycosyltransferase (U3GT) [43,46,47]. However, the complete biosynthetic pathway of steroidal saponins in lilies has not been fully elucidated due to the complex structures of steroidal saponins and their interconversion. Figure 8 shows that the biosynthesis of lily steroidal saponins mainly begins with the dual pathway synthesis of squalene from MVA and MEP pathway, which is catalyzed by SQE to generate 2,3-oxidosqualene, followed by the formation of the tetracyclic steroidal skeleton cycloartenol under the action of CAS. Cycloartenol is then converted to cholesterol through a series of enzymatic reactions. Cholesterol is catalyzed by cholesterol C22-monohydroxylase (CYP90B) to produce 16S,22S-dihydroxycholesterol. 16S,22S-dihydroxycholesterol undergoes cyclization to form the pentacyclic steroidal skeleton 16-hydroxy-22-oxo-cholesterol, and further cyclization generates the hexacyclic steroidal skeleton diosgenin. Diosgenin is a key precursor of various steroidal sapogenins, and its metabolic pathways are branched. Diosgenin can be catalyzed by UGTs to generate funkioside D, or it can be synthesized into (25R)-26-O-(β-D-glu)-furost-5-en-3β,22α,26-triol3-O-α-L-rha-(1→2)-α-L-xyl-(1→3)-β-D-glucopyranoside under the successive action of U3GT and UGTs. In addition, diosgenin can also be hydroxylated to form pennogenin and intermediates A and B. Intermediate A is catalyzed by 3-hydroxy-3-methylglutaryl-CoA (HMG-CoA) to form intermediate C. Finally, intermediates A, B, and C are catalyzed by UGTs to synthesize deacylbrownioside, (25S)-spirost-5-ene-3β,17α,27-triol 3-O-β-D-glu-(1→2)-O-β-D-glu -(1→4)-β-D-glucopyranoside, and lilioglycoside C, respectively. Fan et al. [48] systematically investigated the biosynthetic pathway of steroidal saponins in plants, and Upadhyay et al. [49] reported conversions between steroidal saponin compounds. The biosynthetic framework revealed in these studies is consistent with the proposed biosynthetic pathway of steroidal saponins in *L. lancifolium* bulb in this paper.

### 2.5. Metabolic Pathways for the Biosynthesis of Phenolic Acid Glycerides in L. lancifolium Bulb

**Figure 9:**
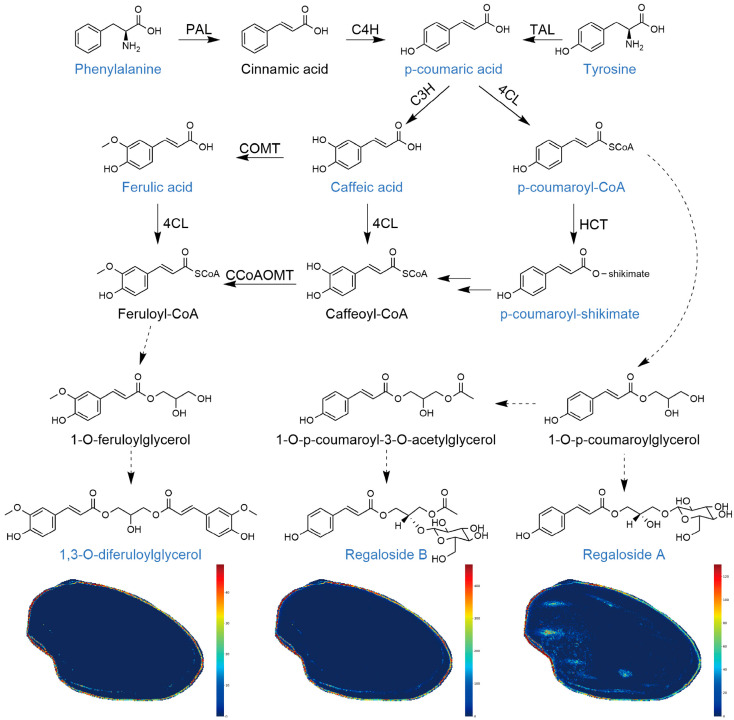
A metabolic pathway for the biosynthesis of phenolic acid glycerides in *L. lancifolium* bulb was proposed based on the integrated analysis of MALDI-MSI and UHPLC-OE-MS. PAL, phenylalanine ammonia-lyase; TAL, tyrosine ammonia-lyase; C4H, cinnamate 4-hydroxylase; C3H, 4-coumarate 3-hydroxylase; 4CL, 4-coumarate-CoA: ligase; COMT, cinnamyl (caffeate) O-methyltransferase; HCT, quinate/shikimate p-hydroxycinnamoyl transferase; CCoAOMT, caffeoyl-CoA 3-O-methyl transferase. The solid black arrows indicate the biosynthetic pathways reported in the literature, while the dashed arrows represent the pathways we propose. Substances labeled in blue font are detected in the present study, while those labeled in black font are not detected in this study.

Phenolic acid glycerides from *L. lancifolium* can be classified into two categories. The first type is an ester compound similar to 1,3-O-diferuloylglycerol, which is formed by the connection between the hydroxyl groups of glycerol molecules and the carboxyl groups of phenolic acids such as caffeic acid and p-coumaric acid. The second type is phenolic acid glycerides, which are further glycosylated to form phenolic acid glycerosides, such as regalosides A and B. Figure 9 shows that this class of compounds is mainly synthesized via the phenylpropanoid metabolic pathway, with two amino acids, namely, tyrosine and phenylalanine, serving as precursors. They are catalyzed by two enzymes, namely, tyrosine ammonia-lyase (TAL) and phenylalanine ammonia-lyase (PAL), to synthesize p-coumaric acid. p-Coumaric acid can be catalyzed by a series of enzymes to synthesize intermediates such as p-coumaric-CoA, feruloyl-CoA, and caffeoyl-CoA. The enzymes involved include 4-coumarate-CoA: ligase (4CL), 4-coumarate 3-hydroxylase (C3H), cinnamyl (caffeate) O-methyltransferase (COMT), caffeoyl-CoA 3-O-methyl transferase (CCoAOMT), and quinate/shikimate p-hydroxycinnamoyl transferase (HCT). Feruloyl-CoA binds to a glycerol backbone to form mono-acylated 1-O-feruloylglycerol, which is then acylated to form 1,3-O-diferuloylglycerol. p-coumaric-CoA links with a glycerol backbone to generate mono-acylated 1-O-p-coumaroylglycerol, which then combines with one molecule of β-D-glucose to synthesize regaloside A. 1-O-p-coumaroylglycerol undergoes hydroxylation to form 1-O-p-coumaroyl-3-O-acetylglycerol, and subsequently connects with one molecule of β-D-glucose to synthesize regaloside B. A study [50] analyzed eight regaloside components in *L. lancifolium* bulbs. The contents of regalosides A and B were significantly higher than those of the other six regaloside components, such as regalosides K and C, which indicates to some extent that regalosides A and B are more enriched in the bulbs. The present study found that the overall spatial distribution density of phenolic acid glycerides in the outer regions (A2, B2) of the bulb was generally higher than that of steroidal saponins. This also indicates that during the bulb maturation process, the metabolic pathway may proceed toward the edge and is more inclined to the biosynthesis of phenolic acid glycerides, especially the synthesis of regaloside components, such as regalosides A and B.

## 3. Materials and Methods

### 3.1. Plant Material

**Figure 10:**
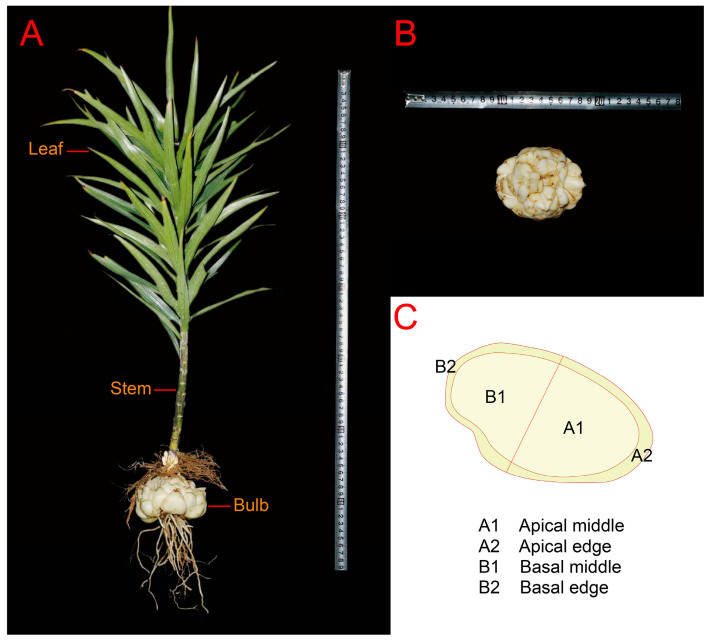
The harvested samples of the *L. lancifolium*: (**A**) the morphological characteristics of the whole plant of *L. lancifolium*; (**B**) the morphological characteristics of the whole bulb of *L. lancifolium*; (**C**) regional division map of MALDI-MSI for an individual bulb scale.

The experimental materials were the mature bulbs of *L. lancifolium* harvested from a lily planting base in Lota Town, Longshan County, Hunan Province, in July 2024. The whole plant measured approximately 69.0 cm in height, and the bulbs were plump (Figure 10A,B). Samples were collected during the optimal harvesting period from June to September, in accordance with the species’ growth cycle. Fresh bulbs were identified on-site by Prof. Qiaozhen Tong from Hunan University of Chinese Medicine as belonging to *Lilium lancifolium* Thunb., a plant in the genus *Lilium* of the *Liliaceae* family. After collection, the surface soil was removed, and the samples were immediately placed in dry ice for transportation and stored at −80 °C in ultra-low temperature conditions until use. During sample preparation, representative individual bulb scales were carefully detached from the middle layer of the bulb, ensuring the exclusion of any damaged or insect-infested parts. Samples were selected from these full and plump scales for research.

### 3.2. Chemicals

Methanol (MeOH), acetonitrile (ACN), and 2-propanol of LC-MS grade were purchased from ANPEL Laboratory Technologies (Shanghai, China) Inc. Acetic acid and α-cyano-4-hydroxycinnamic acid (CHCA) were obtained from Sigma-Aldrich (Darmstadt, Germany). Ammonium hydroxide (NH_4_OH) was purchased from Beijing Bailingwei Technology Co., Ltd. (Beijing, China).

### 3.3. Sample Preparation for MALDI-MSI Analysis

Fresh bulbs should be stored at −80 °C before use. Tissue sectioning was performed on a freezing microtome (Leica CM1950, Nusloch, Germany) at −20 °C to obtain 10 μm thick sections after optimizing the blade angle and sample orientation. The resulting tissue sections were mounted onto indium tin oxide (ITO)-coated glass slides (Bruker, Ettlingen, Germany) with the aid of transfer tape, followed by desiccating in a vacuum dryer for 30 min. To ensure high reproducibility and minimize operator-dependent variation for small-molecule imaging, matrix deposition was executed using an automated HTX TM-Sprayer (HTX-TM Sprayer, Hillsborough, NC, USA). The matrix solution consisted of 10 mg/mL CHCA dissolved in acetonitrile/water (7:3, *v*/*v*) containing 0.1% trifluoroacetic acid. Automated spraying was performed strictly using fixed parameters to maintain inter-slide homogeneity: a nozzle temperature of 75 °C, a flow rate of 100 μL/min, a nitrogen gas pressure of 10 psi, and a nozzle velocity of 1200 mm/min. The substrate was applied to the glass slide four times, with a 10 s drying time between applications. Tissue sections were sprayed using the same matrix preparation and fixed spraying parameters. The matrix coating quality was evaluated by visual inspection after spraying, and representative ion images were examined to exclude obvious streaking, edge effects, or local over-deposition. Three independent biological samples were systematically processed.

### 3.4. MALDI-MSI Analysis

The MALDI-MSI analysis began by fixing ITO glass slides coated with a matrix onto the target plate of the instrument. Target plate height correction and laser focus adjustment were sequentially executed. Prior to acquisition, daily external mass-axis calibration was implemented using an ESI-L Low Concentration Tuning Mix, selecting corresponding positive reference ions across the scanning range. The tissue section detection areas were designated via FlexImaging software (version 7.1, Bruker). All MSI experiments were conducted on a timsTOF FleX mass spectrometry system (Bruker) equipped with a 10 kHz SmartBeam 3D laser operated in positive-ion mode. The laser power was fixed at 65% throughout the experiment, with an imaging spatial resolution set to 30 μm. Mass spectrometry data were acquired over an *m*/*z* range of 50–1200 Da, with each pixel spectrum accumulating 400 laser irradiations. To evaluate ion suppression effects and background chemical interferences, blank matrix (off-tissue) regions were acquired in the same batch and compared with tissue profiles; spectral peaks exhibiting predominant signals in blank areas or presenting homogeneous, non-tissue-associated distributions were excluded from downstream workflows. The MALDI mass spectra were normalized using the root mean square method, and the signal intensity in each image was displayed as the normalized intensity. The tissue sections were scanned in positive-ion mode to obtain the raw mass spectrometry data files (raw data).

### 3.5. Sample Preparation for UHPLC-OE-MS Analysis

The bulb samples were taken out from −80 °C and placed in a freeze dryer (LGJ-10C, Beijing, China) for freeze-drying. Exactly 25 ± 1 mg of the freeze-dried samples were accurately weighed, then mixed with 1000 μL of the extraction solution (MeOH: ACN: water = 2:2:1, *v*/*v*/*v*) and vortexed for 30 s. The mixture was transferred to a homogenizer (JXFSTPRP-24, Shanghai, China) for tissue disruption (35 Hz, 4 min), followed by ultrasonication (PS-60AL, Shenzhen, China) in an ice bath for 5 min. This disruption–ultrasonication cycle was repeated three times. The samples were allowed to stand at −40 °C for 1 h. Subsequently, 200 μL of the supernatant was transferred into a 0.22 μm filter plate. The filter plate–collector plate combination was placed into a positive-pressure device at 4 psi for 90 s. Finally, the filtrate was collected. Five independent biological samples were systematically processed.

### 3.6. UHPLC-OE-MS Analysis

UHPLC-OE-MS analysis was performed using a Vanquish ultra-high-performance liquid chromatography system (Thermo Fisher Scientific, Waltham, MA, USA). Chromatographic separation of the target compounds was achieved on a Phenomenex Kinetex C18 column (2.1 mm × 50 mm, 2.6 μm). The mobile phase consisted of phase A (water containing 0.01% acetic acid) and phase B (isopropanol: acetonitrile, 1:1, *v*/*v*). The elution gradient was established as follows: 0–0.5 min, 1% B; 0.5–4.0 min, 1–99% B; 4.0–4.5 min, 99% B; 4.5–4.55 min, 99–1% B; 4.55–6.0 min, 1% B. The column temperature was maintained at 25 °C, the flow rate was 0.3 mL/min, the sample tray temperature was set at 4 °C, and the injection volume was 2 μL. The experiment used positive- and negative-ion modes of the electrospray ionization source to detect chromatographic effluents with the aim of obtaining mass spectrometry information to the greatest extent to enhance the reliability of substance identification under the proposed analysis conditions. Structural identification was performed based on reversed-phase chromatographic retention behavior, mass spectrometry characteristics, and relevant literature reports. Metabolite identification was performed according to three standard levels: level 1—compounds unequivocally identified by matching RT, MS1, and MS2 with authentic standards; level 2—compounds putatively annotated by matching MS1 and MS2 with public databases; level 3—compounds putatively characterized by matching MS1, MS2, and predicted RT with theoretical databases.

An Orbitrap Exploris 120 (Thermo Fisher Scientific, USA) mass spectrometer was used because of its ability to acquire primary and secondary mass spectrometry data in information-dependent acquisition (IDA) mode under the control of the acquisition software (Xcalibur, version 4.4, Thermo). In this mode, the acquisition software continuously evaluated the full-scan MS spectrum. The detailed parameters are as follows: spray voltage, 3.8 kV (positive-ion mode) or −3.4 kV (negative-ion mode); capillary temperature, 320 °C; vaporizer temperature, 350 °C; sheath gas flow rate, 50 Arb; aux gas flow rate, 15 Arb; sweep gas, 1 Arb; full MS resolution, 60,000; MS/MS resolution, 15,000; and collision energy, SNCE 20/30/40.

### 3.7. Data Analysis

Raw imaging data (raw data) were imported into SCiLS™Lab 2024 software for data processing. The peak intensity was subjected to baseline subtraction, peak alignment, smoothing, and root mean square normalization. The response intensities for the different *m*/*z* values at each spatial point were obtained and converted into pixel points on the imaging map. Comparative inspection of representative ion images before and after normalization confirmed that the baseline spatial localization patterns remained highly consistent. The databases used for identification included the natural product database of RefMetaDB (https://www.biosino.org/RefMetaDB/ (accessed on January 8th, 2025)) and the official website of Reaxys (https://www.reaxys.com/ (accessed on 26 February 2025)).

UHPLC-OE-MS raw data were converted to mzXML format using ProteoWizard software (https://proteowizard.sourceforge.io/, URL (accessed on 15 September 2024)), and then metabolite identification and visual analysis were performed using R packages. All peak intensities were normalized using the total ion chromatogram (TIC), and images were generated using a weak denoising display. The Kyoto Encyclopedia of Genes and Genomes (KEGG, https://www.genome.jp/kegg/ (accessed on 7 March 2025)) was used for metabolic pathway analysis.

## 4. Conclusions

This study marks the first application of MALDI-MSI technology to *L. lancifolium* bulb, successfully achieving in situ, high-resolution spatial visualization of key secondary metabolites, including nine steroidal saponins, three phenolic acid glycerides, and six other metabolites. The analysis clearly revealed that these metabolites were not uniformly distributed throughout the bulb tissue but exhibited basipetal and peripheral spatial distribution patterns. Specifically, most steroidal saponins and phenolic acid glycerides were more enriched in the outer regions (A2, B2) and basal regions (B1, B2) than in the central regions (A1, B1) and apical regions (A1, A2).

Based on these spatial distribution patterns and the integration of four key sterol saponin precursors and seven phenolic acid glyceride precursors identified via UHPLC-OE-MS non-targeted metabolomics, we systematically proposed biosynthetic pathway frameworks for these two classes of characteristic metabolites in *L. lancifolium* bulbs. The visualization-based metabolomics integration strategy established in this study revealed the spatial patterns of metabolite accumulation in *L. lancifolium* bulbs for the first time. It provides crucial theoretical and technical foundations for elucidating their physiological functions, guiding variety selection, and achieving precise utilization of resources.

## Data Availability

The original contributions presented in this study are included in the article and Supplementary Materials. Further inquiries can be directed to the corresponding authors.

## Supplementary Materials

The following supporting information can be downloaded at: https://www.mdpi.com/article/10.3390/molecules31111820/s1, Table S1: The metabolites identified in *L. lancifolium* bulb using MALDI-MSI. Refs. [8,10,51,52,53,54,55,56,57,58,59,60,61,62] are cited in the Supplementary Materials.
