# Supplementary material for: Integrated MALDI-MSI and UHPLC-OE-MS for Spatial Visualization and Biosynthetic Pathway Elucidation of Bioactive Metabolites in Lilium lancifolium Thunb
Source: Molecules. 2026 May 25;31(11):1820. doi: 10.3390/molecules31111820 (PMC13258513; doi:10.3390/molecules31111820)
Supplement: Supplementary file 1 [file molecules-31-01820-s001.zip › molecules-4309711-supplementary.pdf]

Table S1. The metabolites identified in *L. lancifolium* bulb using MALDI-MSI.

Num	Exact_m/z	Cal_m/z	Chemical Name	Formula	Mass error (ppm)	Adduct forms	Reference
**Steroidal saponins**							
**1**	756.4561	756.4528	Deacylbrownioside	$C_{39}H_{62}O_{13}$	4.36	$[M+NH_4]^+$	[1]
	777.3821	777.3822			-0.13	$[M+K]^+$	
**2**	915.4557	915.4584	(25S)-spirost-5-ene-3β,17α,27-triol 3-O-β-D-glu-(1→2)-O-β-D-glu-(1→4) -β-D-glucopyranoside	$C_{45}H_{72}O_{20}$	-2.95	$[M+H-H_2O]^+$	[2]
**3**	742.4363	742.4372	Atropuroside A	$C_{38}H_{60}O_{13}$	-1.21	$[M+NH_4]^+$	[3]
	707.3935	707.4001			-9.33	$[M+H-H_2O]^+$	
	763.3675	763.3666			1.18	$[M+K]^+$	
**4**	754.4409	754.4372	Lilioglycoside C	$C_{39}H_{60}O_{13}$	4.90	$[M+NH_4]^+$	[4]
**5**	918.5046	918.5057	Funkioside D	$C_{45}H_{72}O_{18}$	-1.20	$[M+NH_4]^+$	[3]
	939.4357	939.4351			0.64	$[M+K]^+$	
**6**	633.3002	633.3036	Atropuroside C	$C_{32}H_{50}O_{10}$	-5.37	$[M+K]^+$	[3]
	577.3361	577.3371			-1.73	$[M+H-H_2O]^+$	
**7**	889.4688	889.4792	Pardarinoside E	$C_{44}H_{72}O_{18}$	-11.69	$[M+H]^+$	[5]
**8**	1033.5496	1033.5578	(25R)-26-O-(β-D-glu)-furost-5-en-3β,22α,26-triol 3-O-α-L-rha-(1→2)-α-L-xyl-(1→3)-β-D-glucopyranoside	$C_{51}H_{84}O_{21}$	-7.93	$[M+H]^+$	[6]
**9**	631.3227	631.3243	26-O-β-D-glucopyranosyl nuatigenin	$C_{33}H_{52}O_9$	-2.53	$[M+K]^+$	[1]
**Phenolic acid glycerides**							
**1**	439.0989	439.1001	Regaloside A	$C_{18}H_{24}O_{10}$	-2.73	$[M+K]^+$	[7]
	423.1259	423.1261			-0.47	$[M+Na]^+$	
**2**	481.1106	481.1107	Regaloside B	$C_{20}H_{26}O_{11}$	-0.21	$[M+K]^+$	[7]
	465.1359	465.1367			-1.72	$[M+Na]^+$	
**3**	483.1101	483.1052	1,3-O-diferuloylglycerol	$C_{23}H_{24}O_9$	10.14	$[M+K]^+$	[8]
**Phenylpropanoid**							
**1**	733.1732	733.1741	3,6'-O-diferuloylsucrose	$C_{32}H_{38}O_{17}$	-1.23	$[M+K]^+$	[9]
**Flavonoid**							
**1**	466.1394	466.1344	Astragalin	$C_{21}H_{20}O_{11}$	10.73	$[M+NH_4]^+$	[10]
**Sterol**							
**1**	593.4035	593.4048	Teasterone-3-O-β-D-glucopyranoside	$C_{34}H_{58}O_9$	-2.19	$[M+H-H_2O]^+$	[11]
**Steroidal alkaloid**							
**1**	744.4212	744.4293	β_1-solamargine	$C_{39}H_{63}NO_{11}$	-10.88	$[M+Na]^+$	[12]
**Others**							
**1**	272.0885	272.0895	Hopantenic acid	$C_{10}H_{19}NO_5$	-3.68	$[M+K]^+$	[13]
**2**	279.1104	279.1074	Lilioside E	$C_{11}H_{20}O_9$	10.75	$[M+H-H_2O]^+$	[14]

Annotation: glu: glucopyransoyl; rha: rhamnopyranosyl; xyl: xylopyranosyl.

References

1. Hong, X. X.; Luo, J. G.; Guo, C.; Kong, L. Y. New steroidal saponins from the bulbs of Lilium brownii var. viridulum. *Carbohydr. Res.* **2012,** 361, 19-26.
2. Satou, T.; Mimaki, Y.; Kuroda, M.; Sashida, Y.; Hatakeyama, Y. A pyrroline glucoside ester and steroidal saponins from Lilium martagon. *Phytochemistry* **1996,** 41, (4), 1225-30.
3. Wang, X.; Wu, G. Q. A new steroidal glycoside and potential anticancer cytotoxic activity of compounds isolated from the bulbs of Lilium callosum. *J. Chem. Res.* **2014**, (10), 577-579.
4. Kintya, P. K.; Gur'eva, A. S.; Mashchenko, N. E.; Shashkov, A. S. Structures of some lilioglycosides from the bulbs of Lilium regale. *Chem. Nat. Compd.* **1998,** 33, (6), 658-662.
5. Shimomura, H.; Sashida, Y.; Mimaki, Y. Steroidal saponins, pardarinoside A-G from the bulbs of Lilium pardarinum. *Phytochemistry* **1989,** 28, (11), 3163.
6. Munafo, J. P., Jr.; Ramanathan, A.; Jimenez, L. S.; Gianfagna, T. J. Isolation and structural determination of steroidal glycosides from the bulbs of easter lily (Lilium longiflorum Thunb.). *J. Agric. Food Chem.* **2010,** 58, (15), 8806-13.
7. Qin, Y.; Jin, J.; Zhou, R. R.; Fang, L. Z.; Liu, H.; Zhong, C.; Xie, Y.; Liu, P. A.; Qin, Y. H.; Zhang, S. H. Integrative analysis of metabolome and transcriptome provide new insights into the bitter components of Lilium lancifolium and Lilium brownii. *J. Pharm. Biomed. Anal.* **2022,** 215, 114778.
8. Zhou, Z.; Lin, S.; Yang, H.; Zhang, H.; Xia, J. Antiviral constituents from the bulbs of Lilium lancifolium. *Asian J. Chem.* **2014,** 26, (22), 7616.
9. Kim, B. R.; Thapa, P.; Kim, H. M.; Jin, C. H.; Kim, S. H.; Kim, J. B.; Choi, H.; Han, A. R.; Nam, J. W. Purification of Phenylpropanoids from the Scaly Bulbs of Lilium Longiflorum by CPC and Determination of Their DPP-IV Inhibitory Potentials. *ACS Omega* **2020,** 5, (8), 4050-4057.
10. Chen, W.; Zhang, H.; Wang, J. F.; Hu, X. Flavonoid Glycosides From the Bulbs of Lilium speciosum var. gloriosoides and their Potential Antiviral Activity Against RSV. *Chem. Nat. Compd.* **2019,** 55, (3), 461-464.

11. Soeno, K.; Kyokawa, Y.; Natsume, M.; Abe, H. Teasterone-3-O-beta-D-glucopyranoside, a new conjugated brassinosteroid metabolite from lily cell suspension cultures and its identification in lily anthers. *Biosci., Biotechnol., Biochem.* **2000,** 64, (4), 702-9.
12. Mimaki, Y.; Sashida, Y. Steroidal saponins and alkaloids from the bulbs of Lilium brownii var. colchesteri. *Chem. Pharm. Bull.* **1990,** 38, (11), 3055-9.
13. Zhang, H.; Jin, L.; Zhang, J. B.; Niu, T.; Guo, T.; Chang, J. Chemical constituents from the bulbs of Lilium davidii var. unicolor and anti-insomnia effect. *Fitoterapia* **2022,** 161, 105252.
14. Kaneda, M.; Kobayashi, K.; Nishida, K.; Katsuta, S. Liliosides D and E, two glycerol glucosides from Lilium japonicum. *Phytochemistry* **1984,** 23, (4), 795-798.
